# RNA-Seq Analysis Reveals the Potential Molecular Mechanisms of Puerarin on Intramuscular Fat Deposition in Heat-Stressed Beef Cattle

**DOI:** 10.3389/fnut.2022.817557

**Published:** 2022-03-21

**Authors:** Huan Chen, Tao Peng, Hanle Shang, Xianglong Shang, Xianghui Zhao, Mingren Qu, Xiaozhen Song

**Affiliations:** Jiangxi Province Key Laboratory of Animal Nutrition, Engineering Research Center of Feed Development, Jiangxi Agricultural University, Nanchang, China

**Keywords:** puerarin, beef cattle, heat stress, intramuscular fat deposition, lipid metabolism, RNA-seq

## Abstract

To investigate the effect of *Puerarin* on intramuscular fat deposition in heat-stressed beef cattle and its underlying mechanism. Thirty-two healthy Jinjiang bulls were randomly divided into four groups and dietary with 0 (Control), 200 (Pue200), 400 (Pue400), and 800 (Pue800) mg/kg *Puerarin* in the feed concentrate. The results showed that *Puerarin* treatment enhanced the concentration of crude fat, fatty acid (C14:1 and C17:1), and the activity of fatty acid synthase in *Longissimus thoracis* (LT), but decreased the levels of blood leptin (*P* < 0.05). High-throughput sequencing of mRNA technology (RNA-Seq) was used and the analysis showed that 492 genes were down-regulated and 341 genes were up-regulated in LT, and these genes were significantly enriched to the pathways related to lipid metabolism. These results indicated that dietary supplemental with *Puerarin* enhanced intramuscular fat deposition by regulating lipid metabolism of heat-stressed beef cattle.

## Introduction

Heat stress caused by high temperature is one of the most critical environmental stressors challenging in cattle production (1), which leads to endocrine disorder, abnormal nutrient metabolism and changes in body tissue composition (2, 3). Among the main components of the body, body fat is the most changeable. Many reports have shown that heat stress can inhibit the growth of beef cattle and reduce the deposition of body fat, especially intramuscular fat (4, 5).

Intramuscular fat is one of the main factors used to determine the beef quality grade in many countries due to its beneficial effect on the tenderness, aroma, juiciness, and palatability of beef (6). Intramuscular fat deposition is the result of comprehensive effects of animal growth, body fat distribution, fatty acid composition, key genes of fat metabolism and transcription regulators (7, 8).

*Puerarin* is the main active component of *Pueraria lobata*, and the latter is a traditional Chinese herbal medicine in China that play an important role in relieving muscle, alleviating pain and reducing fever (9, 10). Several reports have demonstrated that *Puerarin* has a protective effect on regulating lipid metabolism, anti-oxidative and anti-inflammation (11, 12). A previous study revealed that *Puerarin*, like estrogen, could affect the hormone secretion levels, thus improve the production performance of animals (13).

Moreover, our previous study has found that *Puerarin* enhanced the immune function and antioxidant capacity of beef cattle in summer, and improved the growth performance and meat quality of heat-stressed beef cattle (14).

However, little attention has been paid to the effect of *Puerarin* on intramuscular fat deposition. In light of the above considerations, the objective of this study was to evaluate the potential efficacy of the supplementation of *Puerarin* on intramuscular fat deposition and analyze its mechanism by RNA-Seq sequencing technology combined with bioinformatics.

## Materials and Methods

### Animal Ethics

All the experimental procedures applied in this study were reviewed and approved by the Committee for the Care and Use of Experimental Animals at Jiangxi Agricultural University (JXAULL-20190015). All procedures involving live animals handling, management, and health care followed the regulations of laboratory animals used for scientific purposes and were implemented within it.

### Puerarin, Animals, and Experimental Design

*Puerarin* was purchased from a company in Xi’an, whose content was 98.1% by analysis of liquid chromatography. The experimental cattle’s feeding and management have been described in detail in our previous study (15). In brief, thirty-two Jinjiang bulls at 15-month-old (291.65 ± 8.84 kg) were randomly divided into four groups (*n* = 8): control group, Pue200, Pue400, Pue800 group (200 mg/kg, 400 mg/kg, and 800 mg/kg *Puerarin* in the feed concentrate), respectively. The composition and nutrient levels of the basal diet were shown in Table 1. The feeding trial lasted for 70 days including a 10-day adaptation period and another 60-day experimental period (July 1 to September 8, and the temperature, relative humidity, and humidity index in cattle house were 30.68°C, 68.05%, and 81.81, respectively).

**TABLE 1:** Composition and nutrient levels of the basal diet (air-dry basis, %).

Ingredients	Content	Nutrient levels	Content
Wheat	56.50	DM	89.42
NaCl	0.50	NE_*mf*_/(MJ/kg)^b^	5.45
NaHCO_3_	1.00	CP	11.19
Premix^a^	2.00	NDF	30.08
Rice straw	20.00	ADF	15.18
Brewer’s grains	20.00	Ash	7.80
Amount	100.00	Ca	1.11
		P	0.67

*^a^The premix provided per kilogram of diet: 3,200 mg of iron as iron sulfate, 1,500 mg of manganese as manganous oxide, 2,000 mg of zinc as zinc oxide, 650 mg of copper as copper sulfate, 35 mg of iodate as calcium iodate, 10 mg of selenium as sodium selenite, 10 mg of cobalt as cobalt chloride, 130 g of calcium as calcium carbonate, 30 g of phosphorus as calcium hydrogen phosphate, 45 mg retinyl acetate, 40 μg cholecalciferol, and 3.0 mg DL-α-tocopheryl acetate. ^b^NE_mf_ were calculated values, while others were measured values.*

### Serum Biochemical Indexes Analysis

On day 60, blood samples (15 mL each, *n* = 6) were collected at 14:00 from the jugular vein and then serum was prepared immediately. The concentrations of serum insulin (INS), triiodothyronine (T3), thyroxine (T4), cortisol (COR), adiponectin (ADPN), and leptin (LEP) were determined by using radioimmunoassay kits (Beijing Sinouk Institute of Biological Technology, China). The levels of total cholesterol (TC), triglyceride (TG), high-density lipoprotein cholesterol (HDL-C), and low-density lipoprotein cholesterol (LDL-C) in serum were measured by using spectrophotometric kits (Nanjing Jiancheng Bioengineering Institute, Nanjing, China).

### Muscle Sample Collection and Analysis

According to the results of blood samples, at the end of the experiment, four bulls with medium body weight were selected from control, Pue400 groups, Pue800 group, respectively. These bulls were transferred to the slaughterhouse and sacrificed at a commercial abattoir following the standard procedures. And then, approximately 200 g of the *longissimus thoracis* (LT) muscle samples from the right half-carcasses between the 12th and 13th ribs were quickly separated, 20 g samples were frozen immediately in liquid nitrogen and stored at −80°C until RNA isolation, and 50 g samples were stored at −20°C for the analysis of the fatty acid composition and the activity of the fatty metabolizing enzyme, other remaining samples were used to determine the contents of moisture, crude protein (CP), crude fat (CF), crude ash (CA), calcium(Ca), and total phosphorus (P) according to Association of Official Analytical Chemists (AOAC). In brief, the content of CA was obtained by incinerating the samples in a muffle furnace at 550°C for 3 h; CP was calculated by quantitative analysis of nitrogen using the Kjeldahl method with copper sulfate and potassium sulfate as catalysts; CF was extracted with diethyl ether using a Soxhlet extractor; Ca and P were determined colorimetrically.

### Fatty Acid Composition and Fatty Metabolizing Enzymes in Muscle Analysis

Briefly, the crude fat in the LT muscle was extracted, and positive hexane, sodium methanol and methyl ester were added in crude fat in turn for fat esterification, and then ethyl acetate was added to obtain fatty acid methyl ester. Fatty acids were expressed as percentages of the total fatty acid methyl esters, which analyzed by a gas chromatograph (Shimadzu, Japan) and the method was referenced to Wang (16). The enzyme activity of fatty acid synthase (FAS), acetyl CoA carboxylase (ACC), hormone sensitive lipase (HSL), and lipoprotein lipase (LPL) in LT muscle of beef cattle were tested using ELISA kits (Delivery code number: ml077321; ml061000; ml061693; ml076623) purchased from Shanghai Enzyme-linked Biotechnology Co., Ltd., (Shanghai, China). Both the in-batch and interbatch coefficients of variation were less than 10%.

### RNA-Seq Library Preparation and Data Analysis

The 60 g of *longissimus thoracis* samples were randomly selected from each of the control and Pue400 groups for RNA-Seq analysis. Total RNA was isolated from eight LT samples from control and Pue400 groups by Trizol reagent (Invitrogen, Waltham, MA, United States) according to the manufacturer’s instructions. The quantity and quality of total RNA were assessed using the Agilent 2100 Bioanalyzer (Agilent, CA, United States). Then, transcriptomic sequencing was performed by Shanghai Majorbio Biopharm Technology Co., (Shanghai, China).

Raw reads of all eight samples were pre-processed through the removal of containing adaptors-read with more than 17% unknown nucleotides. The valid reads of each samples were aligned to the *Bos taurus* genome assembly.^1^

To analyze gene expression, the number of unique-match reads was calculated and normalized to FPKM (Fragment Per Kilo base of exon model per Million mapped reads), which was used to indicate the condition of transcriptional expression. The amount of expression was calculated for each read of the eight sequenced samples by Cuffdiff (17).

To determine the functional categories of differentially expressed genes (DEGs), all DEGs were subjected to GO and KEGG pathway analyses. GO enrichment analysis was used to map all DEGs to GO terms in the GO database.^2^ The significance was calculated using a hypergeometric test by Yang (18).

To better understand the biological function of DEGs, all DEGs were annotated to KEGG (Kyoto encyclopedias of genes and genomes) pathways.^3^

### Quantitative RT-PCR Validation

In order to verify the reproducibility and repeatability of gene expression data obtained by RNA-Seq, seven genes were selected for QRT PCR verification. In brief, cDNA was generated from total RNA using the PrimeScript II 1 st Strand cDNA Synthesis Kit (Takara, Dalian, China) following the manufacturer’s instruction. Quantitative RT-PCR analysis was carried out with the cDNA using SYBR green on a Roche LightCycler 96 real-time PCR machine (Roche, Basel, Switzerland). The b-Actin was used as a reference gene for the standardization of the results. The relative expression levels were calculated as described previously (15). Three biological repeats were measured for each sample. The primers used were shown in Table 2.

**TABLE 2:** Oligonucleotide primers used for quantitative real-time PCR.

Gene name	PrimerName	Sequence (5′-3′)
FABP3	FABP3-F	TGGAGTCGAGTTCGATGAG
	FABP3-R	TTTCCCGCACAAGTGATGTC
ACSL1	ACSL1-F	TACGAAGGCTACGGACAGAC
	ACSL1-R	CCTTGGCAGCCAGGTAATTC
SCD	SCD-F	AGCTGAGAAGCTGGTGATGT
	SCD-R	CAGCGTAACGGAGAAAGGTG
FAS	FAS-F	TGCTGTGCAACTATGCCCTA
	FAS-R	CAGGTGAGGAAGGTGACAGT
HSL	HSL-F	ATCTCCAGCGGACTGGTGTC
	HSL-R	GCACCTGGATCTCGGTGATA
ADPN	ADPN-F	TGGAGAAGGGTGACCAAGTC
	ADPN-R	AAGGAGGAGTCATGGACGTT
FoxO1	FoxO1-F	GTGACATCATGACGCCAGTC
	FoxO1-R	GATGTTGACTGAGCGTGTCC
Actin	Actin-F	TACAATGTGGCCGAGGACTT
	Actin-R	GAGAGAAGGAGGGTGGCTTT

*FABP3, Fatty acid binding protein 3; ACSL1, Long-chain acyl-CoA synthetase 1; SCD, stearoyl-CoA desaturase; FAS, Fatty acid synthase; HSL, Hormone sensitive lipase; ADPN, Adiponectin; and FoxO1, Forkhead transcription factor 1.*

### Statistical Analyses

The serum biochemistry and hormone indexes (*n* = 6) were statistically analyzed by one-way ANOVA with SPSS statistical software (Ver.20 for windows, SPSS), and Tukey–Kramer’s test was used to compare differences among the treatment groups. The muscle nutrients, fatty acid composition, and activity of the fatty metabolizing enzyme of muscle (*n* = 4) were statistically analyzed by *T*-test with SPSS statistical software (Ver.20 for windows, SPSS). All values were expressed as mean ± SE, *P*-value < 0.05 was considered to be significant and 0.05 ≤ *P* < 0.10 was considered as a tendency.

## Results

### Blood Biochemical Characteristics

As presented in Table 3, compared with the control group, dietary supplementation with *Puerarin* by 400 mg/kg and 800 mg/kg decreased the levels of LEP (*P* < 0.001), and the content of TC was reduced in the Pue200 group compared to control and Pue800 (*P* = 0.048). Moreover, the concentration of COR in the Pue400 group was decreased compared with the Pue200 group (*P* = 0.056). No difference was noticed on the contents of INS, T3, T4, ADPN, TG, HDL-C, and LDL-C among all groups.

**TABLE 3:** Effects of puerarin on the blood biochemical characteristics in beef cattle under hot environment.

Item	Groups				*P*-value
	Control	Pue200	Pue400	Pue800	
INS (uIU/ml)	16.02 ± 1.32	16.57 ± 1.32	14.89 ± 2.59	12.69 ± 1.47	0.435
T3 (ng/ml)	1.36 ± 0.07	1.33 ± 0.10	1.20 ± 0.07	1.16 ± 0.16	0.507
T4 (ng/ml)	51.35 ± 3.42	50.69 ± 3.17	47.88 ± 1.09	48.51 ± 2.12	0.749
COR (ng/ml)	48.54 ± 1.72*^ab^*	51.59 ± 2.59*^a^*	45.83 ± 1.33*^b^*	49.43 ± 1.39*^ab^*	0.056
ADPN (mg/L)	14.92 ± 1.38	15.28 ± 1.68	14.51 ± 0.89	14.37 ± 1.03	0.561
LEP (ng/ml)	10.36 ± 0.28*^a^*	9.46 ± 0.70*^a^*	6.91 ± 0.36*^b^*	5.64 ± 0.36*^b^*	<0.001
TC(mmol/L)	4.44 ± 0.15*^a^*	3.68 ± 0.28*^b^*	4.26 ± 0.22*^ab^*	4.54 ± 0.21*^a^*	0.048
TG (mmol/L)	0.34 ± 0.05	0.34 ± 0.03	0.29 ± 0.03	0.35 ± 0.04	0.638
HDL-C(mmol/L)	2.46 ± 0.28	2.20 ± 0.24	2.67 ± 0.24	2.58 ± 0.19	0.545
LDL-C(mmol/L)	0.78 ± 0.13	0.70 ± 0.12	0.69 ± 0.10	0.87 ± 0.16	0.751

*^a,b^Means within a row with no common superscript differ significantly (P < 0.05). All traits in this table were analyzed with cattle as the experimental unit (n:6). INS, insulin; T3, triiodothyronine; T4, thyroxine; COR, cortisol; ADPN, adiponectin; LEP, leptin; TC, total cholesterol; TG, triglyceride; HDL-C, high density lipoprotein cholesterol; and LDL-C, low density lipoprotein cholesterol.*

### Nutritional Components and Fatty Acid Composition of Muscle

As presented in Table 4, compared with the control group, the concentration of CP and CF were increased in the Pue800 group (*P* = 0.039 and *P* = 0.025, respectively). No difference was noticed about the contents of moisture, CA, Ca, and P among all groups. However, dietary supplementation with *Puerarin* by 400 mg/kg enhanced the contents of tetradecenoic acid (C14:1) and heptadecenoic acid (C17:1) compared with the control group (*P* = 0.038 and *P* = 0.020, respectively). Moreover, the Pue400 treatment tended to increase the contents of hexadecenoic acid (C16:1) compared with the control group in Table 5 (*P* = 0.079).

**TABLE 4:** Effect of Puerarin on the nutritional components of muscle in beef cattle under heat stress.

Item	Groups			*P*-value
	Control	Pue400	Pue800	
Moisture/%	71.55 ± 1.38	67.50 ± 2.74	70.68 ± 0.88	0.225
Crude protein/%	20.27 ± 0.43*^b^*	19.54 ± 0.54*^b^*	23.81 ± 0.15*^a^*	0.039
Crude fat/%	4.31 ± 0.40*^b^*	4.67 ± 0.52*^b^*	5.20 ± 0.32*^a^*	0.025
Crude ash/%	4.14 ± 0.16	3.87 ± 0.52	4.15 ± 0.23	0.234
Ca,mmol/g	0.58 ± 0.01	0.56 ± 0.006	0.57 ± 0.005	0.367
P,mmol/g	0.98 ± 0.13	0.87 ± 0.08	0.85 ± 0.01	0.554

*^a,b^Means within a row with no common superscript differ significantly (P < 0.05). All traits in this table were analyzed with cattle as the experimental unit (n:4).*

**TABLE 5:** Effects of Puerarin on fatty acid composition of *longissimus thoracis* muscle beef under heat stress (%).

Items	Groups			*P*-value
	Control	Pue400	Pue800	
C14:0	1.94 ± 0.16	2.63 ± 0.42	2.37 ± 0.34	0.370
C14:1	0.48 ± 0.02*^b^*	0.62 ± 0.03*^a^*	0.56 ± 0.06*^ab^*	0.038
C15:0	0.25 ± 0.04	0.22 ± 0.01	0.26 ± 0.01	0.263
C16:0	20.92 ± 1.35	24.15 ± 1.17	23.93 ± 1.25	0.184
C16:1	2.62 ± 0.21	2.88 ± 0.12	2.49 ± 0.20	0.079
C17:0	0.62 ± 0.07	0.62 ± 0.02	0.69 ± 0.05	0.571
C17:1	0.41 ± 0.01*^b^*	0.51 ± 0.06*^a^*	0.39 ± 0.04*^b^*	0.020
C18:0	17.00 ± 0.88	16.64 ± 0.72	18.21 ± 2.23	0.733
C18:1n9t	0.35 ± 0.05	0.37 ± 0.03	0.29 ± 0.02	0.278
C18:1n9c	38.81 ± 1.19	39.85 ± 1.25	36.75 ± 1.22	0.243
C18:2n6t	0.22 ± 0.02	0.19 ± 0.02	0.18 ± 0.00	0.412
C18:2n6c	2.95 ± 0.28	2.52 ± 0.34	2.84 ± 0.13	0.512
C20:0	0.18 ± 0.01	0.15 ± 0.01	0.17 ± 0.01	0.126
C20:1	0.24 ± 0.07	0.18 ± 0.03	0.19 ± 0.03	0.610
C18:3n3	0.17 ± 0.02	0.14 ± 0.02	0.14 ± 0.01	0.544
Other acids	11.28 ± 1.52	8.29 ± 1.34	10.00 ± 0.14	0.220

*^a,b^Means within a row with no common superscript differ significantly (P < 0.05). All traits in this table were analyzed with cattle as the experimental unit (n:4).*

### The Activity of the Fatty Metabolizing Enzyme

**FIGURE 1:**
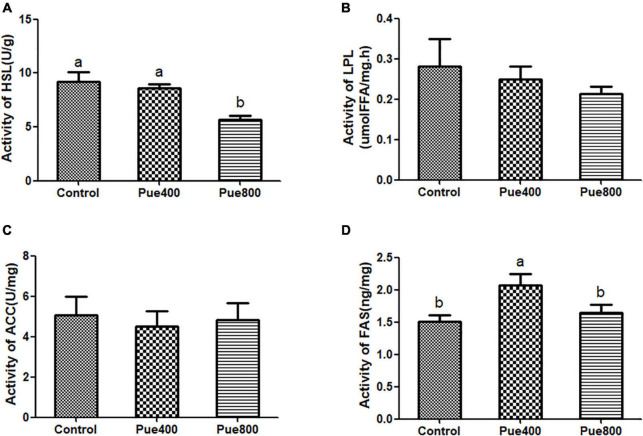
Effect of *Puerarin* on the activity of fat metabolizing enzymes in heat stressed beef cattle (*n* = 4). Control: dietary supplementation with 0 mg/kg *Puerarin* in the feed concentrate; Pue400: dietary supplementation with 400 mg/kg *Puerarin* in the feed concentrate; Pue800: dietary supplementation with 800 mg/kg *Puerarin* in the feed concentrate. HSL, hormone sensitive lipase **(A)**; LPL, lipoprotein lipase **(B)**; ACC, acetyl CoA carboxylase **(C)**; FAS, fatty acid synthase **(D)**. *^a,b^*Means within a row with no common superscript differ significantly (*P* < 0.05).

The results in Figure 1 showed that diet supplemented with 400 mg/kg *Puerarin* improved the activity of FAS in LT (D) (*P* = 0.044), but the activity of HSL in the Pue800 was decreased compared with the control group (A) (*P* = 0.006). No difference was noticed on the activity of LPL and ACC among the three groups.

### Overall Assessment for Mapping Statistics

**FIGURE 2:**
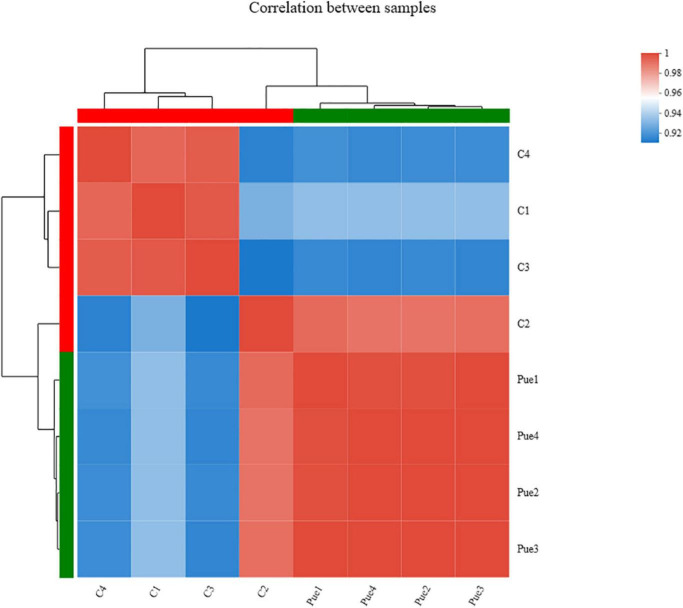
Correlations of eight samples (*n* = 4). C1, C2, C3, C4 were four samples of the Control group (dietary supplementation with 0 mg/kg *Puerarin* in the feed concentrate), and Pue1, Pue2, Pue3, Pue4 were four samples of the Pue400 group (dietary supplementation with 400 mg/kg *Puerarin* in the feed concentrate). In the figure, the right and lower sides are the sample names, the left and upper sides are the sample clustering, and the different color squares represent the correlation of the two samples.

The overall assessment for mapping statistics is shown in Table 6. The RNA-Seq of eight LT samples yielded around 4 billion raw reads. After quality filtering, the high-quality sequence data in each muscle sample was approximately 5.03 gigabases (Gb), ranging from 4.48 to 5.87 Gb. The correlation analysis based on the gene expression profiles found that the correlations between biological replicates were greater than 0.952 (Figure 2), the high reproductivity between samples indicated that the sequencing data could be used for further analyses.

**TABLE 6:** Summary statistics for sequence quality and alignment information of eight *longissimus thoracis* muscle samples in two groups.

Items	Sample name							
	C1	C2	C3	C4	Pue1	Pue2	Pue3	Pue4
Raw reads	48652354	48175450	49644444	50765412	52708852	59764926	45526386	52692342
Clean reads	48120844	47648332	49077762	50105526	52105286	58726390	44767384	51755462
Valid Ratio%	98.91	98.91	98.86	98.70	98.85	98.26	98.33	98.22
Q30(%)	94.87	94.91	94.91	94.56	94.87	93.16	93.73	93.53
GC content(%)	54.16	53.19	53.73	54.00	53.97	53.93	53.88	54.10
Total reads	48120844	47648332	49077762	50105526	52105286	58726390	44767384	51755462
Reads Total mapped	46336007	45862232	47212767	48232193	50036325	55815890	42614063	49308979
Multiple mapped	3273916	4078776	3636977	3706831	4449377	4900461	3691312	4408835
Uniquely mapped	43062091	41783456	43575790	44525362	45586948	50915429	38922751	44900144
Mapping rate (%)	96.29	96.25	96.20	96.26	96.03	95.04	95.19	95.27

*C1, C2, C3, C4 were four samples of the Control group (dietary supplementation with 0 mg/kg Puerarin in the feed concentrate), and Pue1,Pue2,Pue3,Pue4 were four samples of the Pue400 group (dietary supplementation with 400 mg/kg Puerarin in the feed concentrate).*

### Gene Differential Expression Analysis

**FIGURE 3:**
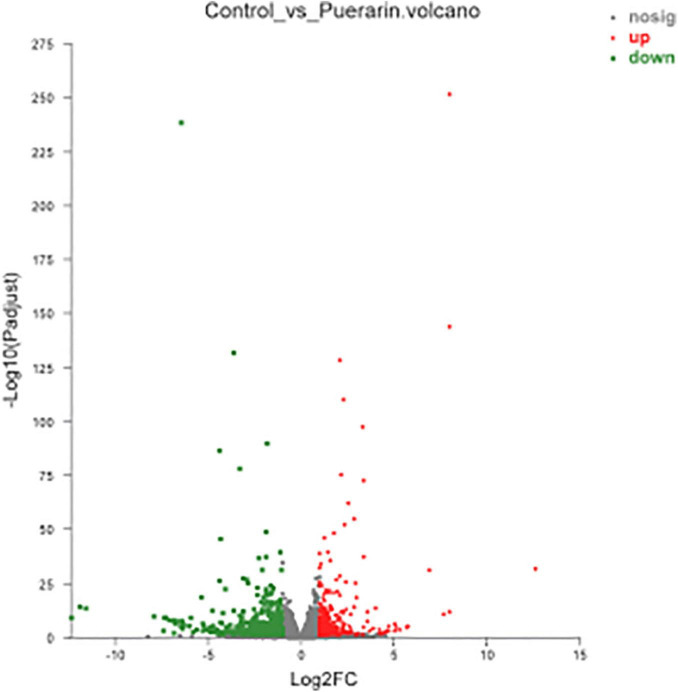
Volcano plot of the differentially expressed genes between control and *Puerarin* groups of *longissimus thoracis* muscles (*n* = 4). Control: dietary supplementation with 0 mg/kg *Puerarin* in the feed concentrate; *Puerarin*: dietary supplementation with 400 mg/kg *Puerarin* in the feed concentrate. The red dots represent the up-regulated DEGs, the green dots represent the down-regulated DEGs and the blue dots represent non-DEGs.

**FIGURE 4:**
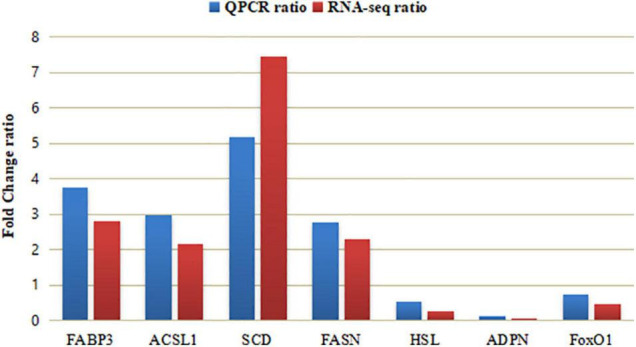
Gene ontology (GO) annotation of differentially expressed genes.

As shown in Figure 3, there were 833 differentially expressed genes (DEGs) were found in LT muscles between the control and *Puerarin* groups, these DEGs were categorized into three gene ontology categories: molecular function, biological process, and cellular component (Figure 4). The top five cellular component categories of DEGs between the control and *Puerarin* groups included “binding,” “catalytic activity,” “molecular function regulator,” “transport activity” and “molecular transducer activity.” The top five biological processes of DEGs included “cellular process,” “biological regulation,” “metabolic process,” “response stimulus” and “developmental process.” The top five cellular components of DEGs included “cell part,” “organelle,” “membrane part,” “membrane” and “organelle part.” Among the total 833 DEGs, 341 DEGs upregulated and 492 DEGs downregulated were identified in the *Puerarin* group compared with the control group.

**FIGURE 5:**
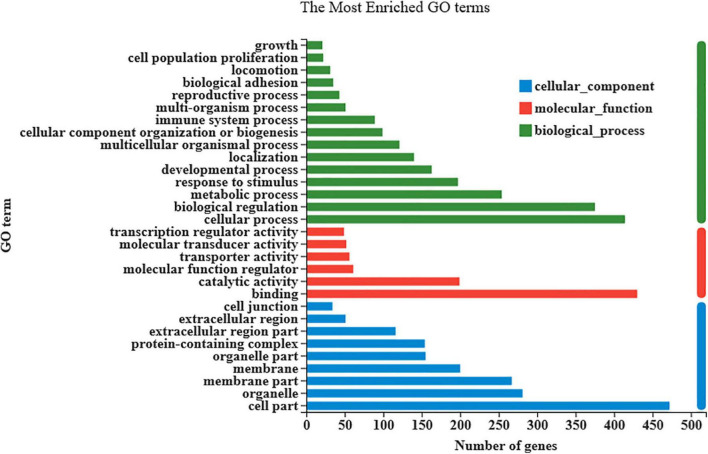
Comparing analysis of relative gene expression in Control and *Puerarin* group (*n* = 4). Control: dietary supplementation with 0 mg/kg *Puerarin* in the feed concentrate; *Puerarin*: dietary supplementation with 400 mg/kg *Puerarin* in the feed concentrate. FABP3, Fatty acid binding protein 3; ACSL1, Long-chain acyl-CoA synthetase 1; SCD, stearoyl-CoA desaturase; FAS, Fatty acid synthase; HSL, Hormone sensitive lipase; ADPN, Adiponectin; and FoxO1, Forkhead transcription factor 1.

There were 20 DEGs related to lipid metabolism, and *Puerarin* treatment enhanced the expression of 15 genes including FATP5, CD36, FABP3, FABP7, ACSL1, Gadd45G, SCD, IRS3, FAS (Table 7). To validate the reliability of the transcriptomic sequencing analyses, 7 differentially expressed genes were randomly selected for qRT-PCR verification (Table 2). As shown in Figure 5, the results from both methods were largely consistent, suggesting that the RNA-Seq results were credible.

**TABLE 7:** Differentially expressed genes related to lipid metabolism.

Gene ID	Gene name	Control	*Puerarin*	*P*-value	Expression trend
Gene18657	FATP5	0.85	1.83	2.14E−07	UP
Gene4568	CD36	0.44	0.97	1.36E−02	UP
Gene1326	FABP3	294.41	822.62	1.39E−03	UP
Gene9621	FABP7	0.69	1.83	1.15E−02	UP
Gene24921	ACSL1	52.49	113.48	2.03E−03	UP
Gene18229	Gadd45G/	1.84	4.09	3.73E−04	UP
Gene24505	SCD	3.67	27.35	1.98E−05	UP
Gene24080	IRS3	0.03	0.78	7.40E−06	UP
Gene19867	FAS	6.90	15.79	2.24E−06	UP
Gene17954	HSL	11.4	2.97	1.86E−03	DOWN
Gene7009	ACOX3	14.02	3.62	2.85E−04	DOWN
Gene470	ADPN	89.61	4.20	5.73E−07	DOWN
Gene4212	LEP	0.42	0.02	4.98E−07	DOWN
Gene14329	ADCY8	0.03	0.11	3.14E−02	UP
Gene16089	PIK3CD	0.45	0.94	2.79E−05	UP
Gene4517	PIK3CG	0.52	1.33	1.81E−03	UP
Gene6887	PRKG2	0.33	0.83	4.50E−04	UP
Gene12812	FoxO1	14.23	6.57	3.12E−03	DOWN
Gene6918	MAPK10	0.02	0.80	1.81E−02	UP
Gene19063	CAMKK1	0.21	0.50	2.38E−02	UP

*Control: dietary supplementation with 0 mg/kg Puerarin in the feed concentrate; Puerarin: dietary supplementation with 400 mg/kg Puerarin in the feed concentrate. FATP5, Fatty acid transporter protein 5; CD36, Fatty acid transposase; FABP, Fatty acid binding protein; ACSL1, Long-chain acyl-CoA synthetase 1; Gadd45G/, Growth arrest and DNA Damage-inducible Protein GADD45 gamma; SCD, stearoyl-CoA desaturase; IRS3, Insulin receptor substrate 3; FAS, Fatty acid synthase; HSL, Hormone sensitive lipase; ACOX3, Acyl-CoA oxidase 3; ADPN, Adiponectin; LEP, Leptin; ADCY8, Adenylate cyclase 8; PIK3CD, Phosphoinositide-3-kinase catalytic delta polypeptide; PIK3CG, Phosphoinositide-3-kinase catalytic gamma polypeptide; PRKG2, Protein kinase cGMP-dependent type II; FoxO1, Forkhead transcription factor 1; MAPK10, Mitogen activated protein kinase 10; and CAMKK1, Calcium/calmodulin dependent protein kin 1.*

Kyoto Encyclopedia of Genes and Genomes (KEGG) pathway enrichment analysis on the downregulated genes demonstrated. There were 36 significantly enriched signaling pathways (*P* < 0.05). As shown in Table 8 in the top 20 significantly enriched signaling pathways, peroxisome proliferator-activated receptor (PPAR) signaling pathway (*P* = 7.73E−07), adenylate activated protein kinase (AMPK) signaling pathway (*P* = 7.22E−05) and forkhead transcription factor (FoxO) signaling pathway (*P* = 1.84E−02) were closely related to lipid metabolism and meat quality in animals.

**TABLE 8:** Classification of DEG according to the KEGG pathways enrichment analysis.

Pathway name	Input number	Background number	*P*-value
PPAR signaling pathway	17	93	7.73E−07
AMPK signaling pathway	18	150	7.22E−05
Regulation of lipolysis in adipocytes	12	65	7.57E−05
Insulin resistance	15	125	4.48E−04
Natural killer cell-mediated cytotoxicity	20	221	8.69E−04
Adipocytokine signaling pathway	11	79	1.26E−03
Phagosome	19	225	2.61E−03
Neuroactive ligand-receptor interaction	25	364	5.19E−03
Type II diabetes mellitus	8	53	5.36E−03
Kaposi sarcoma-associated herpesvirus infection	20	263	5.54E−03
Breast cancer	15	176	8.64E−03
Cellular senescence	17	217	8.85E−03
Human T-cell leukemia virus 1 infection	24	389	1.63E−02
Estrogen signaling pathway	13	154	1.69E−02
Viral carcinogenesis	20	298	1.77E−02
FoxO signaling pathway	13	153	1.84E−02
Influenza A	16	228	2.64E−02
Hepatitis C	16	226	2.69E−02
C-type lectin receptor signaling pathway	10	108	2.71E−02
Endocrine resistance	10	110	2.80E−02

## Discussion

The present study was performed under high temperature and humidity during the summer months (the average THI = 81.81), which indicated that the experimental beef cattle were in a state of heat stress according to the report of Armstrong (19). Studies have shown that heat-stressed can activate the hypothalamic-pituitary-adrenal (HPA) axis, causing a series of complex physiological and metabolic changes, such as elevated level of corticosterone hormone and leptin, which can indirectly reflect the impact of heat-stressed on animals (20). Leptin, an adipokines secreted by adipose, plays an effective role in energy homeostasis, neuroendocrine function and metabolism (21). A study has shown that the concentration of leptin in the blood would increase under heat stress (22). The current results showed that *Puerarin* treatments declined the levels of leptin significantly compared with the control group, which indicates dietary supplementation with *Puerarin* may relieve the disordered endocrine function of beef cattle due to heat stress. *Puerarin* could block the increased levels of the adrenocortico-tropic hormone in the serum, which is induced by single prolonged stress (SPS) (23). Therefore, we can conclude that *Puerarin* can relieve the response of heat stress.

*Puerarin* has been shown to have a direct effect on lipid metabolism in our study. The concentrations of triglyceride (TG) and total cholesterol (TC) in serum can be used as an important index of lipid metabolism, and leptin can inhibit the expression of fatty acid synthase, which is negatively correlated with fat deposition. In this experiment, *Puerarin* treatment with 200 mg/kg decreased the levels of TC, and the levels of leptin in Pue400 and Pue800 groups were significantly lower than those in the control group, which confirmed that *Puerarin* had a direct role in promoting lipid metabolism. Some studies have mentioned that *Puerarin* has a negative effect on animal fat deposition (24, 25). While, other studies have shown that the addition of *Puerarin* enhanced preadipocyte differentiation as well as lipid accumulation (26, 27). The reason for different result may be the treatment concentration of puerarin and the species of laboratory animal.

The content and composition of fatty acids in muscle are closely related to muscle quality (28). Yang found that the content of monounsaturated fatty acids (such as oleic acid and linolenic acid) was correlated with flavor positively, which can help prevent diseases and is beneficial to human health when absorbed unsaturated fatty acids appropriately (29). Tan found that adding isoflavones to the diet can reduce the level of saturated fatty acids in the muscle of the goat, increase the level of monounsaturated fatty acids, and increase the ratio of n-6 to n-3 fatty acids (30). Study has shown that grazing mutton has a better flavor than barn-fed sheep meat, another study found that C14:1 in grazing sheep meat was significantly higher than that in barn-fed sheep meat (31, 32). In this experiment, adding 400 mg/kg *Puerarin* significantly increased the content of C14:1 and C17:1 in muscle, which indicated that *Puerarin* can improve the flavor of postmortem beef.

Fatty acid synthase play an important catalytic role in the synthesis of long-chain fatty acids (33), and HSL is the key enzyme of regulating the rate of lipolysis. In this experiment, the activities of FAS in LT were significantly increased in the Pue400 group, while the activities of HSL were decreased in the Pue800 group, which suggested that *Puerarin* could regulate fat metabolism and promote fat synthesis. These results agree well with the findings of Zhao, which indicated that adding *Daidzein*, a similar structure with *Puerarin*, can affect the lipid metabolism and promote intramuscular fat deposition of Xiangzhong black cattle (34).

Further, to reveal the molecular mechanism of *Puerarin* promoting intramuscular fat deposition, high-throughput sequencing of mRNA technology (RNA-Seq) was performed. The current results showed that *Puerarin* treatment with 400 mg/kg up-regulated 341 DEGs and down-regulated 492 DEGs in LT muscle, and these DEGs are mainly enriched in the PPAR signaling pathway, AMPK signaling pathway, and FoxO signaling pathway. Among them, the PPAR signaling pathway and AMPK signaling pathway are correlated with lipid metabolism and meat quality, and the FoxO signaling pathway is associated with cell-lipid differentiation. The FoxO signaling pathway plays an important role in regulating preadipocyte differentiation (35). After differentiation of preadipocytes, the synthesis and deposition of triglycerides in adipose cells were accelerated, which increased the volume of fat cells. Lu found that FoxO could enhance glucose synthesis and lipolysis (36). Sakamoto suggested that the expression of the Gadd45 gene could be regulated directly by the FoxO signaling pathway, which could promote the differentiation of preadipocytes by participating in DNA methylation in cells (37). Chen found that activated FOXO1 binds to the PPARγ promoter and inhibits the transcriptional activity of PPARγ by competitively suppressing the formation of functional PPARγ/RXR/DNA complex, thereby inhibiting lipogenesis and adipocyte differentiation (38). Our results showed that the expression of FoxO1 genes in the FoxO signaling pathway in the *Puerarin* group was down-regulated significantly and the expression of the Gadd45 gene was up-regulating, which indicated *Puerarin* could promote the differentiation of fat cells in the heat-stressed beef cattle muscles.

Mammalian body contains four important fat depots, namely, visceral, subcutaneous, intermuscular, and intramuscular (IM) fat. But among them, the IM fat is considered one of the most important factors that determines carcass quality traits (39). PUFAs was decreased with the increasing IMF%, which may account for the reduce of PUFAs content in the *Puerarin* group (40). From the view of molecular, the deposition of adipose tissue is essentially the result of spatiotemporal specific expression regulation of many adipose synthesis genes (41). Studies have shown that the PPAR signaling pathway plays a leading role in the process of lipid synthesis, which can directly regulate the expression of SCD, FAS, FABP, GLUT4 and other genes (42, 43), and promote glucose absorption and fat synthesis of adipocytes when it was activated (44).

The PPAR signaling pathway regulates cellular differentiation, energy balance, and lipid metabolism (45). PPAR has three exists isoforms, α, β and γ (46). Furthermore, it was reported that activation of PPARγ is to be essential for deposition of intramuscular fat (47). PPARγ can increase lipid deposition in adipocytes by regulating the levels of expression of HSL, LEP, ADPN and other cytokines produced by adipose tissue (48), and regulating the transcription of a variety of genes involved in fat synthesis, such as FATP, FABP, CD36 (49, 50). After the activation of PPARγ, the levels of expression of FABP3/FABP7, ACSL1, CD36, IRS3, SCD, FAS and other genes in the pathway were significantly up-regulated, and the levels of expression of HSL, LEP, ADPN and other genes were significantly down-regulated. Among the up-regulated genes, FABP is a member of the fatty acid-binding protein family and plays a very important role in the uptake of long-chain fatty acids. ACSL1 can prolong the long-chain fatty acids in cells (51), IRS3 is the receptor of short-chain fatty acids on cell membrane (52), SCD is the main enzyme for *de novo* synthesis of monounsaturated fatty acids (53), FAS is the key enzyme in the process of fatty acid synthesis. Among the down-regulated genes, HSL, LEP and ADPN are the key factors affecting lipolysis. ACSL1 is elevated by PPARγ agonists in the adipose tissue, and ACSL1 overexpression can promote triglyceride accumulation in adipocytes (54, 55). A previous study showed that the higher ACSL1 expression in the F line than the C line coincided with the greater IMF deposition found in the former (56), which was in line with our result. In bovine mammary glands, mRNA abundance at 60 days postpartum of FABP3 and ACSL1 were 80- and 7-fold greater relative to 15 days antenatal, respectively, which are significantly associated with milk fat synthesis (57). Kae found that isoflavone daidzein and its metabolite equol enhance adipocyte differentiation through activating PPARγ (58). Genistein, a main soy isoflavone, can directly bind to and activate peroxisome proliferators-activated receptor a (PPARa) or PPARc (59). Therefore, the activation of PPAR and the expression of its downstream regulatory genes are the most fundamental reason for promoting fat deposition, especially intramuscular fat deposition.

## Conclusion

In a word, *Puerarin* can activate the PPARγ signaling pathway, up-regulate the levels of expression of genes related to fat synthesis, and down-regulated genes expression promoting muscle fatty acid oxidation, so as to regulate lipid metabolism, improve the beef flavor of Jinjiang cattle and enhance intramuscular fat deposition in LT muscle of heat-stressed beef cattle.

## Data Availability Statement

The datasets presented in this study can be found in online repositories. The names of the repository/repositories and accession number(s) can be found in the article/supplementary material.

## Ethics Statement

The animal study was reviewed and approved by the Committee for the Care and Use of Experimental Animals at Jiangxi Agricultural University (JXAULL-20190015).

## Author Contributions

XSo, HC, and TP designed the overall study. HS, XSh, XZ, and MQ performed the animal feeding experiment and sample analysis. XSo and HC wrote the manuscript. All authors contributed to the article and approved the submitted version.

## Conflict of Interest

The authors declare that the research was conducted in the absence of any commercial or financial relationships that could be construed as a potential conflict of interest.

## Publisher’s Note

All claims expressed in this article are solely those of the authors and do not necessarily represent those of their affiliated organizations, or those of the publisher, the editors and the reviewers. Any product that may be evaluated in this article, or claim that may be made by its manufacturer, is not guaranteed or endorsed by the publisher.

## Footnotes

1
ftp://ftp.ensembl.org/pub/release-75/fasta/bos_taurus/dna/

2
http://geneontology.org

3
https://www.genome.jp/kegg/pathway.html
